# Hair and urinary 2-hydroxynaphthalene levels in the people living in a region with frequent oil pipeline incidents in Iran: Health risk assessment

**DOI:** 10.1371/journal.pone.0308310

**Published:** 2024-09-06

**Authors:** Sara Hemati, Mohsen Heidari, Fariborz Momenbeik, Abbas khodabakhshi, Abdolmajid Fadaei, Marzieh Farhadkhani, Fazel Mohammadi-Moghadam

**Affiliations:** 1 Department of Environmental Health Engineering, School of Health, Shahrekord University of Medical Sciences, Shahrekord, Iran; 2 Department of Environmental Health Engineering, Faculty of Medical Sciences, Tarbiat Modares University, Tehran, Iran; 3 Department of Chemistry, University of Isfahan, Isfahan, Iran; 4 Assistant Professor of Environmental Health, Educational Development Center, Shahrekord University of Medical Sciences, Shahrekord, Iran; Center for Research and Technology Transfer, VIET NAM

## Abstract

Oil spills from pipeline accidents can have long-lasting health effects on residents of polluted regions. Assessing the potential health risk of these accidents is crucial for effective environmental health management. This study analyzed the concentration of 2-OHNAP in urine and hair as biomarkers of PAHs exposure among the people living in a region with frequent oil pipeline incident in Iran. Fifty pairs of hair and urine samples were collected from residents along with demographic information and dietary habits via a questionnaire. The concentration of 2-OHNAP was analyzed using high performance liquid chromatography coupled with fluorescence detector (HPLC-FLD). 2-OHNAP was detected in 100% of urine and 88% of hair samples. The mean concentration of 2-OHNAP in urine was 16.65 ± 21.98 μg/g creatinine and in hair was 8.16±7.62 ng/g dry weight (dw). However, there was no significant correlations between the levels of 2-OHNAP in urine and hair. The mean values of HQ and CR were below 1 and 10^−6^, respectively. Moreover, some simulated health risk indices were near the threshold levels, and the carcinogenic risk above 70% of the simulated CRs was above 10^−6^ as well. Therefore, the health risk attributed to the exposure to the parent compound of 2-OHNAP in the study area is currently acceptable, but it is not negligible and may be worsened in the future. This study provides a valuable scientific information for regional decision makers and stakeholders about human health programs and identification of environmental health priorities.

## Introduction

Over the last few decades, there has been a significant growth in the discovery and extraction of crude oil around the world, which has increased the risk of inadvertent discharge into the environment [1, 2]. The environmental leakage of crude oil and its fractions has resulted in a huge number of contaminated areas [3, 4]. The issue of environmental pollution caused by crude oil and derivatives has emerged as a significant global concern due to its substantial contribution to ecological and social harm [3–5].

Polycyclic aromatic hydrocarbons (PAHs) are ubiquitous and can be found in both natural and anthropogenic sources, such as incomplete combustion of fossil fuels, fuel leaks, coke production, traffic exhaust, forest fires and microbiological leakage [6, 7]. PAHs have attracted considerable interest due to their widespread presence and their toxicological potential [8, 9]. These compounds have been found in a variety of environmental matrices such as water, air, soil, sediment and food [10–12]. Human populations can encounter health risks from PAHs generally through ingestion, inhalation and dermal absorption [13, 14]. PAHs have been listed as priority control pollutants by the US Environmental Protection Agency (EPA) due to their long-range transport properties and bioaccumulation [15, 16]. Naphthalene is quantitatively the main component in the PAH-mixture and classified by International Agency for Research on Cancer (IARC) as possibly carcinogenic in humans (group 2B) [17]. Additionally, naphthalene is known to cause acute hemolysis and can cause skin irritation and hypersensitivity [18].

Despite evidence of harmful health effects from PAHs, exposure is nevertheless widespread, and there are no restrictions limiting the quantities of PAHs in the human body and biological matrices [19]. After entering the body, the liver mostly breaks down PAHs into hydroxy-PAHs (OH-PAHs), which have higher genotoxicity than the parents [20, 21]. The metabolites of PAHs are eliminated from the human body by urine and faeces, with an average half-life of less than 30 hours. OH-PAHs have the ability to function as endocrine disruptors, causing detrimental effects on the endocrine system [22, 23]. It is critical to monitor PAHs and their metabolites in environmental and biological samples in order to estimate human exposure to PAHs [23, 24]. Urine is an extremely useful biomonitoring matrix for assessing the internal exposure of humans to PAHs [11]. However, due to the short half-lives of PAHs, the urinary concentration of PAHs and OH-PAH reflect the recent exposure during a certain period [8, 11, 25]. Many studies have proven that human hair can be a helpful biomarker of PAH exposure. In fact, this is due to the low metabolic activity of hair tissue, its stability, and easier collection of hair samples [23, 26].

The most common source of PAHs pollution in Sar Khun city (a non-industrial city with a pristine nature in Chaharmahal and Bakhtiari province, Iran) is petrogenic source arising from accidental spillage of crude oil due to aging pipelines and landslide. Hence, biomonitoring of PAHs and their matabolites is very important environmental health policy to monitor the levels of exposure in PAHs-contaminated regions.

The authors are not aware of any studies on Sar Khun city hydrocarbon contamination and health risk assessment at the time of this study. Therefore, this study investigates the internal exposure levels in the concentrations of naphthalene metabolite (2-hydroxynaphthalene, 2-OHNAP) in Sar Khun city by collecting human urine and hair samples. This is the first study that assesses both hair and urinary OHPAHs in the people lived in this highly polluted area. The presented results will expand the knowledge of public exposure to OHPAHs pollutants in Sar Khun city; as such, the results are a valuable base for more research on the relationship between OHPAHs and human health.

## Materials and methods

### Chemicals and reagents

2-naphthol [2NAP, (MW: 144.17 g/mol, purity 99%)] and β-glucuronidase/sulfatase were obtained from Sigma-Aldrich Inc., (St Louis, MO). Internal standard (hexamehylbenzene) were purchased from the Supelco, USA. HPLC-grade acetonitrile, sodium acetate (reagent grade, > 99%) and all other chemicals and reagents were purchased from Merck (Germany). All stock and working standards were stored at −20°C until further use.

### Study area and population description

Wear and tear of the crude oil pipeline to the refinery and landslide caused this pipeline to break in 2020 in Sar Khun city, Chaharmahal and Bakhtiari province, Iran. This incident led to a large fire along the river and the entry of a significant amount of crude oil into the Sar Khun spring, river and surrounding lands, and caused significant damage to fisheries, agricultural lands, drinking water resources, and horticulture in the region. It is estimated that about 30,000 barrels of crude oil were imported into the region due to a broken pipe. Before this incident, this pipeline was damaged and leaked in 1977, 1982, 2011 and 2016 years.

This study was established between 2022 and 2023, comprising 50 residents aged 18–70 years who had been living in Sarkhoon region for more than 10 years. In this study, inclusion criteria for participants were as follows: (i) without metabolic disease and urinary problems; (ii) Residence in Sar khun city; (iii) without history of occupational exposure to PAHs. People who dyed or permed their hair in the last 2 years, smokers, pregnant women and users of medications were excluded from the study. In addition, 4 people living in areas far from the oil pipeline were selected as controls.

### Biological sample collection

The morning urine samples were collected in 50 mL polypropylene tubes, stored immediately in a refrigerator. Approximately 3 g of hair (2 cm, starting from head scalp) was cut using stainless steel scissors and then wrapped in aluminum foil. The collected urine and hair samples were transferred to the laboratory and kept at −80°C until further analysis. The recruitment period for this study commenced on 24 July 2023 and concluded on the same day. The hair and urine samples were collected in the same days. Moreover, every urine samples creatinine content was immediately determined.

### Consent form

All participants were informed about the objectives and methods of the study before the investigation. This study did not include minors. Each participant signed a- written informed consent form after understanding the purpose of the study and completed a questionnaire that included demographic information and lifestyle variables. The study was approved by the Ethics Committee of Shahrekord University of medical sciences (IR.SKUMS.REC.1401.027). Sample preparation and analysis.

### Urine samples

The urine samples were extracted and cleaned up according to CDC method with some changes [27]. Urine samples (1 mL) were thawed and equilibrated to room temperature. Then diluted with 500 μL of sodium acetate buffer (2 M) and adjusted to pH 5.0. The mixture was fortified with internal standard hexamehylbenzene before adding 10 μL of glucuronidase/arylsulfatase enzyme. The prepared solution was incubated at 37°C for 16–18 h. The 2-OHNAP was purified by extraction with 8 mL of hexane. Following that, the samples were mixed for 10 seconds at 20 rpm, then centrifuged for 10 min at 3000 rpm. The extraction method was repeated twice. The combined hexane extracts were concentrated to complete dryness under a gentle stream of nitrogen and reconstituted with 150 μL acetonitrile.

### Hair samples

Hair samples were extracted and analyzed according to Wang et.al (2020) method [28]. In brief, prior to processing, ultrasonically washed hair samples were pretreated using Triton X-100 surfactant. The digestion process was performed using tetramethylammonium hydroxide solution (TMAH) in ultrasonic cleaner. Dichloromethane and n-hexane (2:3, by volume) were used to extract 2-OHNAP from hair samples. The extraction process was repeated three times and the organic phase was separated by centrifugation at 2000 rpm for 5 min. Finally, the organic phase was completely dried using gentle stream of nitrogen (99.999%) and switched to 100 μL of acetonitrile.

### HPLC-FLD analysis

The 2-OHNAP was analyzed by an Azura high performance liquid chromatography coupled with fluorescence detector (HPLC-FLD). A C18 column (4.6 × 250 mm, 5 μm) was used for analysis. The mobile phase consisted of acetonitrile and water (50:50 v/v) and the flow rate was set at 1 mL min^−1^. Run time was 25 minutes. The excitation /emission wavelength were 227 nm and 355 nm, respectively. The injection volume was set at 20 μL.

### Quality assurance and quality control

The limit of detection (LOD) values for measuring 2-OHNAP in urine and hair were 0.2 μg/L and 0.2 ng/g, respectively. A total of 10 urine samples were analyzed for the evaluation of method precision. Concentrations that were lower than the LOD, were reported as not detected (ND). For health risk assessment and statistical analysis, NDs were substituted by one-half of the detection limit. The limit of quantitation (LOQ) of urine and hair was 1.17 μg/L and 1.17 ng/g respectively. The relative standard deviation (RSD) for replicate analyses of samples was 3.26%. The accuracy of the analytical method was higher than 78.0%. The linearity of the calibration curve was assessed by a linear regression with R^2^ values greater than 0.99 for both urine and hair samples. The valid concentrations of 2-OHNAP were normalized to the urinary creatinine and reported as μg/g creatinine in order to eliminate the effect of urine dilution.

### Risk assessment

In the present study, exposure to PAHs was evaluated by an internal dose approach. Considering that individuals who do not smoke are primarily exposed to PAHs through the dietary intake [29], the evaluation of PAH exposure was only focused on dietary intake. In this regard, the urinary levels of 2-OHNAP were converted to the oral estimated daily intake (EDI) of the parent compound. EDI (mg/kg.day) was calculated in men, women, and all participants. Moreover, EDI was calculated based on the original 2-OHNAP concentrations (EDI_O_) and the concentrations were justified by creatinine (EDI_c_) as follows:

$${EDI}_{O}=\frac{C_{u}\times V_{u}\times {MW}_{P}}{f\times BW\times {MW}_{m}}$$

$${EDI}_{c}=\frac{C_{s}\times C_{c}\times {MW}_{P}}{f\times {MW}_{m}\times 1000}$$

Where C_u_ is urinary 2-OHNAP concentration (μg/L); V_u_ is the urine volume excreted per day (L/day); BW is the body weight (kg); MW_p_ and MW_m_ are the molecular weights of naphthalene and 2-OHNAP, respectively (g/mol); f is the ratio of 2-OHNAP excreted in urine to the total exposure dose (dimensionless); C_s_ is the 2-OHNAP normalized by creatinine concentration (μg/g), and C_c_ is the total urinary creatinine excreted within 24 h standardized by body weight (mg/kg.day). The values of the parameters are presented in Table 1.

**Table 1:** Parameters used for health risk assessment [33].

Parameter	Unit	Value
Body weight, BW	kg	Men: 71.4±14.2 Women: 62.8±8.4 All: 67.1±12.3
Molecular weight of 2-OHNAP, MW_p_	g/mol	144.17
Molecular weight of naphthalene, MWm	g/mol	128.17
Urine volume excreted per day	L/day	2
Urine excretion ratio of PAH, f	%	100
Daily excretion of urinary creatinine, Cc	mg/kg-bw/day	Male: 23 Female: 18
Reference dose, RfD	μg/kg-bw/day	20
Cancer slope factor, CSF		
Toxic equivalency factor of naphthalene, TEF	-	0.001

Non-carcinogenic risk was assessed by calculating Hazard Quotient (HQ) as follows:

$$HQ=\frac{EDI}{RfD}$$

Where reference dose (RfD) is the reference dose (μg/kg-bw/day) [30–32].

The carcinogenic risk (CR) attributed to the exposure with naphthalene, as parent compound of 2-OHNAP, was calculated using the following formulas:

$${EDI}_{B[\alpha ]P_{eq}}=TEF\times EDI$$

$$CR={EDI}_{B[\alpha ]P_{eq}}\times CSF$$

Where ${EDI}_{B[\alpha ]P_{eq}}$ is the B[a]P equivalent of EDI for naphthalene (mg/kg-bw/day); TEF (dimensionless) is the toxic equivalency factor of naphthalene; and CSF is the cancer slope factor of B[a]P ((mg/kg-bw/day)^− 1^) [11].

According to the USEPA’s recommendations, an HQ value of less than 1 indicates no significant potential health risk [34], a CR ranging from 1 × 10^− 6^ to 1 × 10^− 4^ are acceptable or tolerable CR, and the risk lower than 1 × 10^− 6^ considered negligible [35].

In this study, health risk indices were simulated in Crystal Ball software (version 11.1.2.4, Oracle, Inc., USA) based on Monte Carlo simulation (MCS) with 10^5^ iterations. The distributions of the simulated HQ and CR values are presented along with their 5^th^ (P5) and 95^th^ (P95) percentiles, and mean values.

### Statistical analysis

Data were statistically analyzed by IBM SPSS 24.0 (IBM SPSS, NY, USA) at a 95% confidence level. Before selecting the statistical tests, the normality of data was checked with the Kolmogorov-Smirnov Test. The statistical differences between the mean (or median) values of independent groups were tested with independent t-test (or Mann-Whitney U test) and ANOVA (or Kruskal Wallis). Correlations between OH-PAHs concentrations in hair and urine were also performed using Spearman correlation. As noted previously, for statistical analysis, NDs were substituted by one-half of the detection limit.

## Results and discussion

### Demographic characteristics of the study population

The socio-demographic and other characteristics of the study population are shown in Table 2. The proportions of males (50%) and females (50%) were similar. The body mass index (BMI) of 52% of the subjects was between 18.5 and 24.9 kg/m^2^, indicating a normal weight, and 48% of the subjects were overweight. In total, participants had a mean (±SD) age of 46.62±12.87 years and a mean (±SD) BMI of 25.16±4.2 kg/m^2^. Of the all subjects, 54% were uneducated and only 16% of men were unemployment.

**Table 2:** Demographic characteristics of the study population (n = 50).

Characteristics	Mean ± SD or Numbers (%)
**Age, years**	46.62±12.87
**Gender**	
*Male*	25
*Female*	25
**BMI, kg/m** ^**2**^	25.16±4.2
**Urine Creatinine, mg/dl**	144.326
**Occupation status**	
*Employed*	52
*Unemployed*	48
**Education**	
*Uneducated*	54
*Junior school and below*	22
*Senior school*	14
*High school and above*	10

### 2-OHNAP concentration in urine and hair

Concentrations of 2-OHNAP are presented in Table 3 for all subjects. The levels of 2-OHNAP were adjusted by the concentration of urinary creatinine. 2-OHNAP was detected in 100% and 88% of the urine and hair samples, respectively (S1 Table). This suggested the wide exposure of the general public in Sar Khun city to the petroleum-related PAHs. The urinary levels of 2-OHNAP of all subjects ranged from 0.11 to 103.79 μg/g creatinine, with a mean value of 16.65 ± 21.98 μg/g creatinine. The concentrations of 2-OHNAP in hair for all subjects were ranged from 1.02 to 47.11 ng/g dry weight (dw).

**Table 3:** Concentrations of 2-OHNAP among the participants (n = 50).

2-OHNAP	Urine		Hair (ng/g)
	Uncorrected concentration (μg/L)	Creatinine-corrected (μg /g Creatinine)	
Mean ± SD	20.38±25.94	16.65±21.98	8.16±7.62
Median	7.6	6.06	6.68
Min	0.23	0.11	<LOD
Max	89.07	103.79	47.11

Naphthalene, being among the most prevalent PAHs in the environment, has the potential to produce urinary naphthalene metabolites in higher concentrations [36]. The domination of 2-OHNAP was also found in other similar studies in urine samples [19, 37, 38].

The molecular weight of OH-PAHs has an inverse relationship with their concentration in urine [39]. Due to differences in size and composition, metabolites of low molecular weight PAHs are primarily eliminated through urine, while metabolites of bigger PAHs are mostly eliminated through faeces [39, 40].

A comparison of the 2-OHNAP data in urine and hair to the reported concentrations in previous studies is shown in S2 Table. Some data exist for Portuguese, U.S., Korean, Chinese, German and Afghan pediatric populations, but comparison is not always possible due to lack of standardization to creatinine concentration. In the present study, levels of urinary 2-OHNAP were generally found to be higher than that in other countries, such as Poland [41], Italy [39], Canada [8] and the USA [42]. Nevertheless, one study carried out in South China on E-waste recycling workers [26] reported higher urinary levels of 2-OHNAP than those obtained in this research.

There have been few research on the determination of OH-PAHs in human hair. The most abundant concentration for 2-OHNAP was observed in the hair of Chinese women (29.53 ng/g dw) [43] and French children (5.56 ng/g dw) [44]. In the Lin et al study, 2-OH-Nap with the frequency of 71% was detected in hair samples (mean of 14.6 ng/g dw) [26].

Although urine metabolites are commonly used to measure PAHs exposure. Previous research has found that urine PAH metabolite concentrations vary greatly over time due to their rapid elimination kinetics. The metabolism of PAHs in human urine is rapid, and the results can only be seen in the urine within 6–30 hours of exposure [28]. Furthermore, pollutants that enter the human body can enter the hair through blood capillaries [26, 28]. Cell membranes, as an impermeable barrier, can prevent organic ions of medium molecular mass from passing freely into the matrix of hair cells [26, 45]. Hence, hair could serve as an optimal biomaterial for accurately assessing prolonged human exposure to PAHs. However, more reliable data is needed from numerous comparative investigations with other human metabolites.

### OH-PAHs in the urine and hair of subgroups

As noted previously, the participants in this study have different demographic status in views of gender, age and BMI. In order to investigate the factors that affect the PAHs exposure, we conducted an additional analysis on the association between demographic parameters and 2-OHNAP concentrations (S3 Table). In this study, concentrations of 2-OHNAP in urine and hair were compared based on sex, age and BMI.

### Gender

In this study, the levels of 2-OHNAP in both urine and hair samples of females (mean value in urine: 22.43±25.44 μg/g creatinine and hair: 10.44±9.63 ng/g) were higher than those of males (mean value in urine: 10.87±16.44 μg/g creatinine and hair: 6.08±4.41 ng/g). This finding could be explained by gender differences in PAH absorption and metabolism [46].‏ ‏

In this context, Li et al. (2008) found that mean urinary 2-OHNAP concentrations in females in the United States (2.64 ng/mL) were somewhat higher than in males (2.19 ng/mL) [47]. In contrast, the average levels of 2-OHNAP in Iranian males were 1.8 times greater than in females [36]. Despite the difference in the 2-OHNAP concentrations ‏of urine and hair between females and males, Mann-Whitney test showed no significant differences in OH-PAH concentrations (p-values of 0.118 for urine and 0.260 for hair). Similarly, several studies have reported such insignificant difference in the 2-OHNAP between females and males [48–50].‏ Overall, further study is needed to establish valid findings about the effect of gender on PAH residues in human urine and hair.

### Age

The spearman rank correlation showed no significant correlation between 2- OHNAP concentrations and age (r = 0.071 and p-value = 0.626 for urine, and r = 0.252 and p-value = 0.077 for hair). With respect to the low half-life of OH-PAHs in the human body (< 30 h) and the ready excretion of them via urine, these metabolites are not bioaccumulated in the body. Moreover, other authors reported no associations between urinary OHPAHs and age [43, 48] while one study finds the positive association [51]. This result should thus be confirmed on a population with wider age range.

### BMI

Obesity can affect the concentration of PAHs metabolites in urine due to the fact that PAHs are chemicals that dissolve in fat. Thus, the correlations between OH-PAHs and BMIs were investigated. In this current study, no significant differences were detected between the OH-PAH concentrations and BMI (p>0.05). The results of other study are inconsistent with the present study. There were notable variations in OH-PAH levels among subjects based on BMI (p < 0.01) in the study conducted by Huang et al. (2022). Based on these findings, obesity may influence exposure of PAHs. This correlation between OHPAHs and BMI has been previously reported by other researchers [18, 52], indicating the ability of PAHs to dissolve in fat [51]. This finding should be replicated in a larger sample with a broader age range.

### The relationship between the 2-OHNAP in hair and urine

Spearman rank correlation showed that there was insignificant correlation between the concentrations of 2-OHNAP in hair and urine (r = 0.037, p = 0.799). Many investigations indicate that parent PAHs undergo two biotransformation phases in the human body. The parent PAHs are metabolized into OH-PAHs during the first phase. During the second phase, they are rapidly detoxified to glucuronide and sulphate. The subsequent metabolites are primarily eliminated via urine and faeces [25, 26].

Lin et al. (2020) discovered that metabolites of PAHs were eliminated from the body either as independent molecules or in a combined form [26]. Furthermore, OH-PAHs concentrations in human urine indicate recent exposure due to its short half-life. The half-life of 2-OHNAP is between 4.9 and 12.2 hours. In addition, urine samples are typically collected in a random manner, which can be affected by short-term fluctuations in exposure [42]. However, because of its slow growth rate, hair is a good bioindicator of long-term exposure. Consequently, the levels of OH-PAHs in urine did not correspond to those in hair, leading to non-significant correlation [28, 43].

### Potential sources of PAHs exposure

In present study, the detection of a high level of 2-OHNAP in urine and hair indicates the pollution of the studied area with petroleum compounds. As previously mentioned, this region has experienced multiple instances of crude oil spills resulting from a ruptured oil pipeline. The level of pollution is so high that among the 50 participants, 37 confirmed smelling oil near the local river. Moreover, it is crucial to emphasize that there is no industrial activity either within the studied area or in its vicinity, rendering this area untouched or pristine. The 2-OHNAP concentrations in the urine and hair samples collected from a neighbor area, without the oil pollution (mean value in urine: 0.18±0.09 μg/g creatinine and hair: 0.7±0.08 ng/g), was far lower than the studied area (mean value in urine: 16.65±21.98 μg/g creatinine and hair: 8.16±7.62 ng/g) with significant statistical difference (P<0.001). Previous studies have shown that in the areas with no strength point source of pollution, diet is considered as the primary source of human exposure to PAHs in non-smokers with more than 70% contribution [39, 51].

Among the foodstuff meat is associated with increased urinary levels of naphthalene metabolites. Indeed, food processing procedures such as grilling or smoking increase the PAHs content [21]. It is noteworthy that grilled meat is one of the most consumed foods in the diet of the study area. Before sample collection, 43 participants in the study area and all participants in the background area stated that they consumed grilled meat during last 72 hr. The low levels of 2-OHNAP in the samples collected from background area, despite consuming grilled meat, reveal that consuming of grilled meat could not cause such high levels of 2-OHNAP in the polluted area. Moreover, none of the participants were smoker. Therefore, it can be asserted that the 2-OHNAP metabolite in the body of residents in the studies area has been stemmed from the environmental pollution due to the numerous pipeline fractures in recent years. It can be also concluded that during 2 years after the last oil spill accident, the various environmental matrices in the study area has not been completely remediated.

### Health risks of 2-OHNAP exposure

In order to assess the body burden and health risks of the parent compound of 2-OHNAP, naphthalene, EDI and risk indices were calculated in a probabilistic procedure using Monte-Carlo simulation method. The EDI was estimated based on the urinal concentrations of 2-OHNAP and several exposure parameters, and the P5th, mean and P95th values are presented in S4 Table. According to this Table, the mean values of EDI for females, males, and all participants in the study area were 32.9, 21.7, and 27.4 μg/day, which are higher than those reported for populations in South China (5.93 μg/day), Japan (6.8 μg/day), India (8 μg/day) and Vietnam (11 μg/day), and Malaysia (3.8 μg/day) [26, 53].

**Fig 1:**
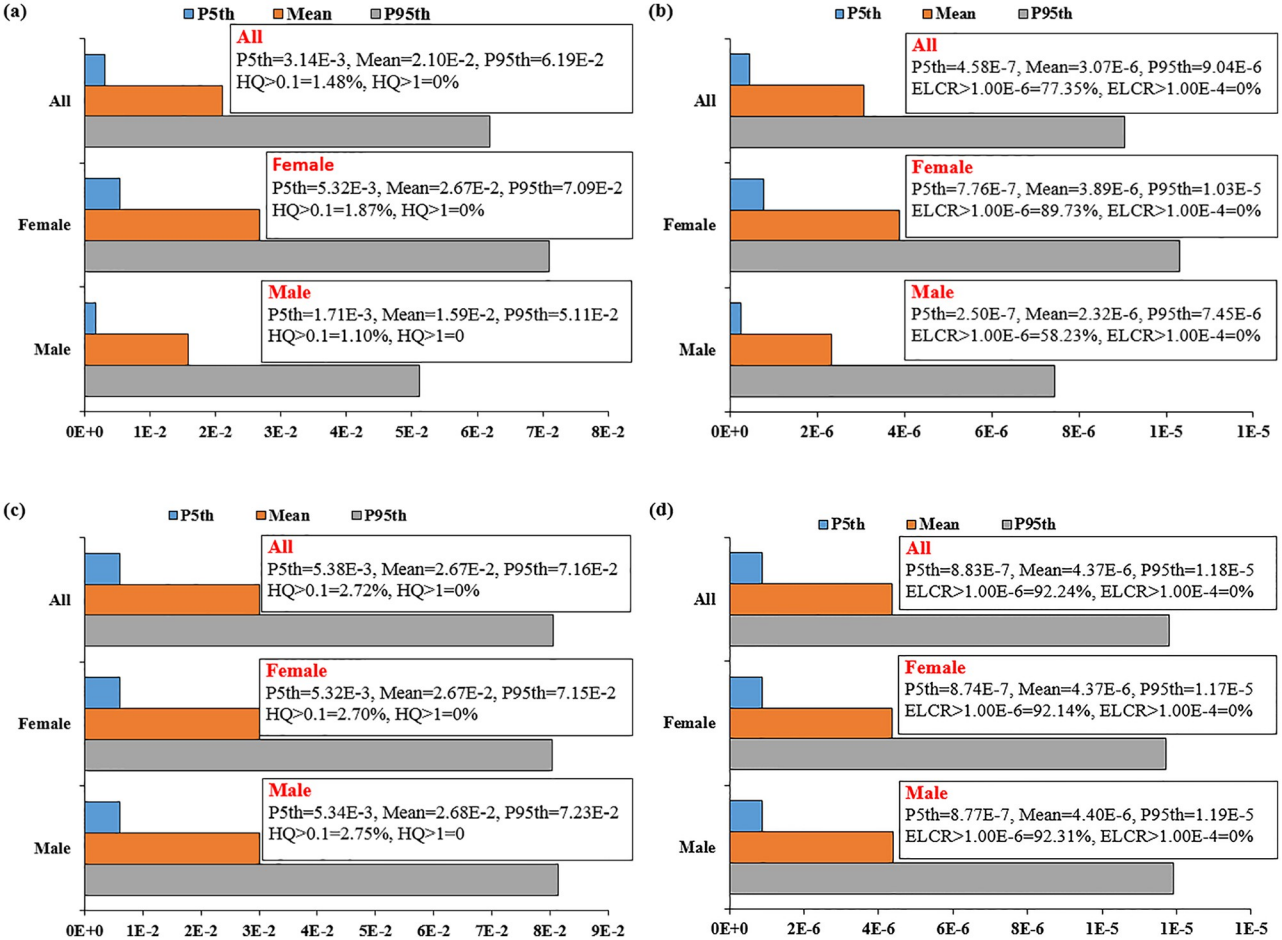
Probabilistic health risks based on original urinal 2-OHNAP concentrations (a: non-carcinogenic, b: carcinogenic), and Creatinine-adjusted procedure (c: non-carcinogenic, d: carcinogenic).

By considering the RfD of 20 μg/kg-bw/day for naphthalene and the EDI vales, non-carcinogenic risk (HQ) was calculated and the simulated values for 10^5^ iterations are presented in Fig 1. The mean values of HQ were below 1, indicating that there is no significant adverse health effect to people in studied region. However, the HQ values were not negligible, as around 2% of the simulated HQs were above 0.1. Therefore, the non-carcinogenic risks attributed to the parent compound of 2-OHNAP should not be overlooked and any increase in the environmental pollution, especially just after a new accident or other exposure parameters may result in the unacceptable risks levels (Fig 1a).

By integrating the EDI, TEF and CSF inputs, the carcinogenic risks attribute to the parent compound of 2-OHNAP was calculated and the simulated CR values are presented in Fig 1b. According to USEPA, the CR values <10^−6^ and >10^−4^ are negligible and unacceptable, respectively. As is evident from Fig 1b, all the simulated CR values were below 10^−4^ with mean value of 3.07E-6. However, 77.35% of data were above 10^−6^, indicating that the CR attributed to the intake of the parent compound of 2-OHNAP is not negligible. This result is in good agreement with other published papers in regarding 2-OHNAP health risk [11, 30, 31, 54].

Regarding the higher urinal concentration of 2-OHNAP and lower weight of women compared to men, the higher levels of EDI and health risks for women was expectable. Accordingly, the mean values of HQ and ELCR for women were higher than those of men, but all of the simulated HQ and ELCR values for women was below 1 and 10^−4^, respectively. Note that, in this study, we also calculated the health risks adjusted by creatinine (Fig 1c and 1d). Previous studies has mentioned that the average the creatinine excretion rate normalized by the body weight, C_c_ (mg/kg-bw/day) [33, 55]. Since the proposed Cc for men (23 mg/kg-bw/day) is higher than women (18 mg/kg-bw/day), the estimated EDI (S4 Table), and health indices (Fig 1) for both genders were similar. Overall, the risk indices attributed to the intake of naphthalene, as a typical pollutant related to environmental oil pollution, were in the safe levels, but their values were not in the negligible ranges. As noted previously, the last oil accident in the studied area was occurred around 2 years before conducting this study. Certainly, a new accident may result in very high levels of the oil-related PAHs, which may result in high carcinogenic and non-carcinogenic risks. Therefore, taking into account that humans are exposed to a mixture of PAHs, and in the present study the cumulative risk was assessed for only 2-OHNAP, there may be an underestimation of the risk. Therefore, policymaker in the study area should take especial attention to the safety of the oil pipeline located in Sarkhun region.

Finally, monitoring of contaminants like 2-OHNAP in water, food, and soil is crucial for effective safeguarding public health and managing environmental health risks. Regular monitoring helps the detection of the presence and concentration of harmful substances, enabling timely interventions to prevent exposure. By identification of contaminant hotspots, authorities can implement targeted remediation efforts and regulatory measures to mitigate risks. In regions with frequent oil pipeline incidents, such as the studied area in Iran, the extent of contamination can be significant and widespread. Persistent contaminants can remain in the environment for extended periods, affecting soil health, water quality, and food safety.

### Strength and limitations

Our study had several major strengths. All participants were selected from the general community and did not have any occupational exposure to PAHs. We focused our analysis on non-smokers and measured the metabolite simultaneously in human hair and urine. A major limitation of this investigation was the small sample sizes, which hindered our ability to identify statistically significant relationships. A greater sample size would provide more reliable statistical results in future research. The metabolite was analyzed in first-morning urine samples, which reflects the short-term exposure level of 2-OHNAP because of the short half-lives of PAHs in the human body. However, we also analyzed the 2-OHNAP level in hair, which indicates long-term exposure. Moreover, in this study, we have focused on one metabolite of oil-related PAHs, and it was revealed that the risks attributed to the parent compound of this metabolite alone was not negligible.

## Conclusions and future prospects

This work is the first to report OH-PAH levels in a region with frequent oil pipeline incident in Iran. In the present research, 2-OHNAP was detected in 100% and 88% of the urine and hair samples of residents living in crude oil polluted area, respectively. However, no significant correlations of OH-PAH concentrations between hair and urine were found. According to our data, the level of exposure to 2-OHNAP in the region were relatively high compared to other areas. Health risk assessments results indicated that the risks of human exposure to PAHs may be underestimated if exposure is only limited to the levels of 2-OHNAP in urine and hair. However, further research is needed to confirm our findings. However, the non-carcinogenic risks attributed ‏to the parent compound of 2-OHNAP should not be overlooked and any increase in the ‏environmental pollution, especially just after a new accident, or other exposure pathways may ‏result in the unacceptable risk levels. ‏The results found in this study indicate the relevance of urine and hair analysis for the PAHs biomonitoring and their metabolites in the studied region. These findings unveiled that as well as urgent responses, effective science communication for impacted societies must be considered by governments and federal organizations.

## Supporting information

S1 Table Concentrations of 2-OHNAP among the participants (n = 50). (DOCX)

S2 Table Comparison of the 2-OHNAP levels in urine and hair among different countries around the world. (DOCX)

S3 Table Spearman correlation coefficients between demographic characteristics and 2-OHNAP in hair and urine. (DOCX)

S4 Table EDI of the parent compound of 2-OHNAP in the study area population. (DOCX)

## Abbreviations

2NAP: 2-Naphthol
2-OHNAP: 2-hydroxynaphthalene
BMI: Body Mass Index
CR: Carcinogenic Risk
EDI: Estimated Daily Intake
EPA: Environmental Protection Agency
HPLC-FLD: High Performance Liquid Chromatography coupled with Fluorescence Detector
HQ: Hazard Quotient
IARC: International Agency for Research on Cancer
LOD: Limit of Detection
LOQ: Limit of Quantitation
OH-PAHs: Hydroxy-PAHs
PAHs: Polycyclic aromatic hydrocarbons
RSD: Relative Standard Deviation
TMAH: Tetra Methyl Ammonium Hydroxide
