# Supplementary material for: Hair and urinary 2-hydroxynaphthalene levels in the people living in a region with frequent oil pipeline incidents in Iran: Health risk assessment
Source: PLoS One. 2024 Sep 6;19(9):e0308310. doi: 10.1371/journal.pone.0308310 (PMC11379380; doi:10.1371/journal.pone.0308310)
Supplement: S1 Table — (DOCX) [file pone.0308310.s001.docx]

**S1 Table.** Concentrations of 2-OHNAP among the participants (n=50)

| #Num | Gender | Age | BMI (Kg/m^2^) | Urine Creatinine (mg/dl) | 2-OHNAP in urine (µg/L) | 2-OHNAP (Creatinineµg/g) | 2-OHNAP in hair (ng/g) |
| --- | --- | --- | --- | --- | --- | --- | --- |
| 1 | Female | 54 | 23.2 | 88.8 | 27.98 | 31.51 | 7.32 |
| 2 | Male | 64 | 31.1 | 74.5 | 3.67 | 4.92 | 7.30 |
| 3 | Male | 51 | 23.2 | 83.4 | 2.90 | 3.47 | 3.25 |
| 4 | Male | 44 | 25.7 | 165.7 | 0.78 | 0.47 | 3.96 |
| 5 | Male | 34 | 16.5 | 156.4 | 7.84 | 5.01 | 3.37 |
| 6 | Male | 56 | 36 | 69.5 | 0.23 | 0.33 | 9.45 |
| 7 | Male | 53 | 30.8 | 121.8 | 76.19 | 62.55 | 3.88 |
| 8 | Male | 31 | 26.8 | 177.5 | 35.31 | 19.89 | 2.14 |
| 9 | Male | 27 | 21.5 | 107.2 | 2.57 | 2.40 | 3.38 |
| 10 | Male | 68 | 27.4 | 59.6 | 0.87 | 1.45 | ND |
| 11 | Female | 39 | 23 | 155.8 | 0.80 | 0.51 | 3.51 |
| 12 | Male | 50 | 27.5 | 105.8 | 4.21 | 3.98 | 1.40 |
| 13 | Female | 45 | 21.9 | 213.3 | 4.38 | 2.05 | 47.11 |
| 14 | Female | 50 | 22.5 | 156.5 | 8.59 | 5.49 | 9.92 |
| 15 | Female | 44 | 30.7 | 103.3 | 1.95 | 1.89 | 6.29 |
| 16 | Female | 31 | 26.2 | 161.3 | 1.12 | 0.69 | 3.17 |
| 17 | Female | 43 | 26.2 | 76.7 | 0.69 | 0.89 | 1.60 |
| 18 | Male | 55 | 26 | 137.3 | 7.05 | 5.14 | 1.02 |
| 19 | Female | 54 | 24.6 | 182.3 | 89.07 | 48.86 | 18.32 |
| 20 | Female | 45 | 24.5 | 89.9 | 19.59 | 21.79 | 13.55 |
| 21 | Male | 25 | 22.3 | 264.2 | 5.02 | 1.90 | 5.01 |
| 22 | Female | 61 | 22.9 | 177.3 | 17.52 | 9.88 | 15.57 |
| 23 | Female | 50 | 28.5 | 56.2 | 20.19 | 35.93 | 12.58 |
| 24 | Female | 47 | 32 | 123.6 | 79.34 | 64.19 | ND |
| 25 | Male | 64 | 26.6 | 85.1 | 12.35 | 14.51 | 5.32 |
| 26 | Male | 60 | 28.7 | 115.9 | 44.34 | 38.25 | 15.46 |
| 27 | Female | 42 | 20.6 | 108.9 | 13.11 | 12.04 | 1.40 |
| 28 | Female | 50 | 21.9 | 147 | 52.88 | 35.97 | ND |
| 29 | Male | 22 | 24.8 | 304.5 | 65.46 | 21.50 | ND |
| 30 | Female | 46 | 34.4 | 90.8 | 10.93 | 12.04 | ND |
| 31 | Female | 53 | 25.7 | 57.3 | 59.47 | 103.79 | ND |
| 32 | Male | 41 | 17.7 | 98.4 | 0.65 | 0.66 | 4.02 |
| 33 | Female | 68 | 24.6 | 95 | 10.51 | 11.06 | 5.13 |
| 34 | Female | 43 | 28.5 | 117.9 | 57.46 | 48.74 | 5.84 |
| 35 | Female | 19 | 25.1 | 194 | 0.75 | 0.39 | 2.76 |
| 36 | Female | 32 | 20 | 78.1 | 12.94 | 16.57 | 12.38 |
| 37 | Male | 58 | 26.1 | 222.5 | 2.31 | 1.04 | 8.20 |
| 38 | Female | 62 | 22.7 | 96.6 | 49.05 | 50.78 | 10.85 |
| 39 | Male | 46 | 21.3 | 125.2 | 2.35 | 1.88 | 2.21 |
| 40 | Male | 26 | 18.7 | 95 | 6.90 | 7.26 | 10.14 |
| 41 | Female | 27 | 24.9 | 254.1 | 68.05 | 26.78 | 9.79 |
| 42 | Male | 42 | 22.6 | 106 | 10.94 | 10.33 | 1.36 |
| 43 | Male | 29 | 28.1 | 148.6 | 76.59 | 51.54 | 7.07 |
| 44 | Male | 43 | 22.3 | 100.2 | 3.72 | 3.71 | 16.02 |
| 45 | Female | 50 | 26.3 | 135.5 | 22.62 | 16.70 | 9.80 |
| 46 | Male | 57 | 31.6 | 111.1 | 7.36 | 6.63 | 10.32 |
| 47 | Male | 33 | 24.1 | 253.4 | 6.11 | 2.41 | 2.96 |
| 48 | Male | 62 | 24.5 | 116.1 | 0.67 | 0.58 | 12.48 |
| 49 | Female | 62 | 28.7 | 716 | 0.78 | 0.11 | 13.21 |
| 50 | Female | 63 | 16.5 | 135.2 | 2.87 | 2.12 | 9.21 |
