# Supplementary material for: Hair and urinary 2-hydroxynaphthalene levels in the people living in a region with frequent oil pipeline incidents in Iran: Health risk assessment
Source: PLoS One. 2024 Sep 6;19(9):e0308310. doi: 10.1371/journal.pone.0308310 (PMC11379380; doi:10.1371/journal.pone.0308310)
Supplement: S2 Table — (DOCX) [file pone.0308310.s002.docx]

**S2 Table.** Comparison of the 2-OHNAP levels in urine and hair among different countries around the world

| # Study | Country | # Samples | | Mean | | Population | Analytical technique | Reference |
| --- | --- | --- | --- | --- | --- | --- | --- | --- |
|  |  | Urine | Hair | Urine (μg/g creatinine) | Hair (ng/g dw) |  |  |  |
| 1 | Canada | 97 | - | 7.3 | - | Combined (children, young adults, elders) | GC-MS | [1] |
| 2 | Czech Republic | 330 | - | 7.5 | - | Mothers | HPLC–MS/MS | [2] |
| 3 | South China | 27 | 27 | 18.1 | 16.1 | E-waste recycling workers | HPLC-MS | [3] |
|  |  | 29 | 29 | 15 | 19.1 | Non-e-waste recycling workers |  |  |
|  |  | 21 | 21 | 16.4 | 18.8 | Adult residents |  |  |
| 4 | USA | 515 | - | 2.7 | - | Healthy volunteers | HPLC–MS/MS | [4] |
| 5 | China | 102 | - | - | 29.53 | Healthy women | GC-MS/MS | [5] |
| 6 | Czech Republic | 531 | - | 5.5 | - | Mothers and their newborn children | HPLC–MS/MS | [6] |
| 7 | United States | 1768 |  | 4.3 | - | Adult women | GC-MS/MS | [7] |
| 8 | Italy | 394 | - | 4.637 | - | Adult population in the area of waste incinerator | HPLC–MS/MS | [8] |
| 9 | France | - | 19 | - | 5.56 | Children | GC-MS/MS | [9] |
| 10 | Poland | 218 | - | 8.291 | - | Children | GC-MS | [10] |
| 11 | Iran | 222 | - | 3.07 | - | General population | GC-MS | [11] |
| 12 | Germany | 516 | - | 3.706 | - | Children and adolescent | HPLC–MS/MS | [12] |
| 13 | Iran | 50 | 50 | **16.65** | **8.16** | Adult residents | HPLC-FLD | Present study |

**References**

1. Ratelle, M., et al., Polycyclic aromatic hydrocarbons (PAHs) levels in urine samples collected in a subarctic region of the Northwest Territories, Canada. Environmental research, 2020. 182: p. 109112.

2. Urbancova, K., et al., Comparison of polycyclic aromatic hydrocarbon metabolite concentrations in urine of mothers and their newborns. Science of the Total Environment, 2020. 723: p. 138116.

3. Lin, M., et al., Insights into biomonitoring of human exposure to polycyclic aromatic hydrocarbons with hair analysis: A case study in e-waste recycling area. Environment international, 2020. 136: p. 105432.

4. Zhu, H., M.-P. Martinez-Moral, and K. Kannan, Variability in urinary biomarkers of human exposure to polycyclic aromatic hydrocarbons and its association with oxidative stress. Environment international, 2021. 156: p. 106720.

5. Palazzi, P., et al., Exposure to polycyclic aromatic hydrocarbons in women living in the Chinese cities of BaoDing and Dalian revealed by hair analysis. Environment international, 2018. 121: p. 1341-1354.

6. Urbancova, K., et al., Evaluation of 11 polycyclic aromatic hydrocarbon metabolites in urine of Czech mothers and newborns. Science of the Total Environment, 2017. 577: p. 212-219.

7. Guo, J., et al., Associations of urinary polycyclic aromatic hydrocarbons with bone mass density and osteoporosis in US adults, NHANES 2005–2010. Environmental Pollution, 2018. 240: p. 209-218.

8. Iamiceli, A.L., et al., Biomonitoring of the adult population in the area of turin waste incinerator: baseline levels of polycyclic aromatic hydrocarbon metabolites. Environmental research, 2020. 181: p. 108903.

9. Palazzi, P., E.M. Hardy, and B.M. Appenzeller, Biomonitoring of children exposure to urban pollution and environmental tobacco smoke with hair analysis–a pilot study on children living in Paris and Yeu Island, France. Science of the Total Environment, 2019. 665: p. 864-872.

10. Sochacka-Tatara, E., et al., Urinary polycyclic aromatic hydrocarbon metabolites among 3-year-old children from Krakow, Poland. Environmental research, 2018. 164: p. 212-220.

11. Hoseini, M., et al., Environmental and lifestyle factors affecting exposure to polycyclic aromatic hydrocarbons in the general population in a Middle Eastern area. Environmental Pollution, 2018. 240: p. 781-792.

12. Murawski, A., et al., Polycyclic aromatic hydrocarbons (PAH) in urine of children and adolescents in Germany–human biomonitoring results of the German Environmental Survey 2014–2017 (GerES V). International journal of hygiene and environmental health, 2020. 226: p. 113491.
