# Supplementary material for: Hair and urinary 2-hydroxynaphthalene levels in the people living in a region with frequent oil pipeline incidents in Iran: Health risk assessment
Source: PLoS One. 2024 Sep 6;19(9):e0308310. doi: 10.1371/journal.pone.0308310 (PMC11379380; doi:10.1371/journal.pone.0308310)
Supplement: S3 Table — (DOCX) [file pone.0308310.s003.docx]

S3 Table. Spearman correlation coefficients between ‎demographic characteristics and 2-OHNAP ‎in hair and urine

| Influencing Factors | 2-OHNAP concentration | | | |
| --- | --- | --- | --- | --- |
|  | Urine | | Hair | |
|  | r | p | r | p |
| Weight | 0.109 | 0.449 | -0.132 | 0.362 |
| BMI | 0.137 | 0.342 | -0.079 | 0.587 |
| Age | 0.071 | 0.626 | 0.252 | 0.077 |
