# Supplementary material for: Hair and urinary 2-hydroxynaphthalene levels in the people living in a region with frequent oil pipeline incidents in Iran: Health risk assessment
Source: PLoS One. 2024 Sep 6;19(9):e0308310. doi: 10.1371/journal.pone.0308310 (PMC11379380; doi:10.1371/journal.pone.0308310)
Supplement: S4 Table — (DOCX) [file pone.0308310.s004.docx]

S4 Table. EDI of the parent compound of 2-OHNAP in the study area population

| **Procedure** | **Gender** | **EDI** | **P5th** | **Mean** | **P95th** |
| --- | --- | --- | --- | --- | --- |
| Original Data | Female | µg/d | 6.80E+0 | 3.29E+1 | 8.77E+1 |
|  |  | µg/kg/d | 1.07E-1 | 5.34E-1 | 1.43E+0 |
|  | Male | µg/d | 2.47E+0 | 2.17E+1 | 6.81E+1 |
|  |  | µg/kg/d | 3.44E-2 | 3.19E-1 | 1.02E+0 |
|  | All | µg/d | 4.33E+0 | 2.74E+1 | 7.97E+1 |
|  |  | µg/kg/d | 6.32E-2 | 4.25E-1 | 1.26E+0 |
| Creatinine-adjusted procedure | Female | µg/d | 4.33E+00 | 2.05E+01 | 5.28E+01 |
|  |  | µg/kg/d | 6.88E-02 | 3.26E-01 | 8.40E-01 |
|  | Male | µg/d | 1.45E+00 | 1.62E+01 | 5.02E+01 |
|  |  | µg/kg/d | 2.04E-02 | 2.27E-01 | 7.02E-01 |
|  | All | µg/d | 2.51E+00 | 1.87E+01 | 5.23E+01 |
|  |  | µg/kg/d | 3.73E-02 | 2.78E-01 | 7.79E-01 |
